# Streamlined Quantification of p-γ-H2AX Foci for DNA Damage Analysis in Melanoma and Melanocyte Co-cultures Exposed to FLASH Irradiation Using Automated Image Cytometry

**DOI:** 10.21769/BioProtoc.5208

**Published:** 2025-02-20

**Authors:** Stefana Orobeti, Ioana Dinca, Alexandra Bran, Ion Tiseanu, Felix Sima, Stefana M. Petrescu, Livia E. Sima

**Affiliations:** 1 Department of Molecular and Cellular Biology, Institute of Biochemistry, Bucharest, Romania; 2 National Institute for Laser, Plasma, and Radiation Physics, Bucharest-Magurele, Romania

**Keywords:** Cancer cells, FLASH LPA electrons cell irradiation, DNA damage, Immunofluorescence, γ-H2AX foci, Image cytometry

## Abstract

In response to DNA-damaging physical or chemical agents, the DNA damage repair (DDR) pathway is activated in eukaryotic cells. In the radiobiology field, it is important to assess the DNA damage effect of a certain irradiation regime on cancer cells and compare it to the effect on non-transformed cells exposed to identical conditions. The first step in the DNA repair mechanism consists of the attachment of proteins such as the phosphorylated histone γ-H2AX (p-γ-H2AX) to DNA double-strand breaks (DSB) in the nucleus, which leads to the formation of repairing foci. Therefore, imaging methods were established to evaluate the presence of foci inside the nucleus after exposure to DNA-damaging agents. This approach is superior in sensitivity to other methods, such as the comet assay or the pulsed-field gel electrophoresis (PFGE), that allow direct detection of cleaved DNA fragments. These electrophoresis-based methods require high ionizing radiation dosages and are difficult to reproduce compared to imaging-based assays. Conventionally, the number of foci is determined visually, with limited accuracy and throughput. Here, by exploring the effect of laser-plasma accelerated electrons FLASH irradiation on cancer cells, we describe an image cytometry protocol for the quantification of foci with increased throughput, upon large areas, with increased precision and sample-to-sample consistency. It consists of the automatic scanning of fluorescently labeled cells and using a gating strategy similar to flow cytometry to discriminate cells in co-culture based on nuclei elongation properties, followed by automatic quantification of foci number and statistical analysis. The protocol can be used to monitor the kinetics of DNA repair by quantification of p-γ-H2AX at different time points post-exposure or by quantification of other DNA repair proteins that form foci at the DNA DSB sites. Also, the protocol can be used for quantifying the response to chemical agents targeting DNA. This protocol can be performed on any type of cancer cells, and our gating strategy to discriminate cells in co-culture can also be used in other research applications.

Key features

• Analysis of DNA-damage sensitivity using model cancer cell lines and non-transformed cellular controls.

• Allows comparative testing of various doses of DNA damaging radiation on cancer and non-transformed cells in co-culture, as well as in monocultures.

• This protocol requires TissueFAXSiPlus model i12 or an alternative instrument that allows automatic image acquisition and stitching to benefit from enhanced analysis throughput.

• For analyses of co-cultures or heterogeneous samples, TissueQuest software is required to selectively quantify different cell subpopulations; dedicated training is advisable before operating the system.

## Graphical overview


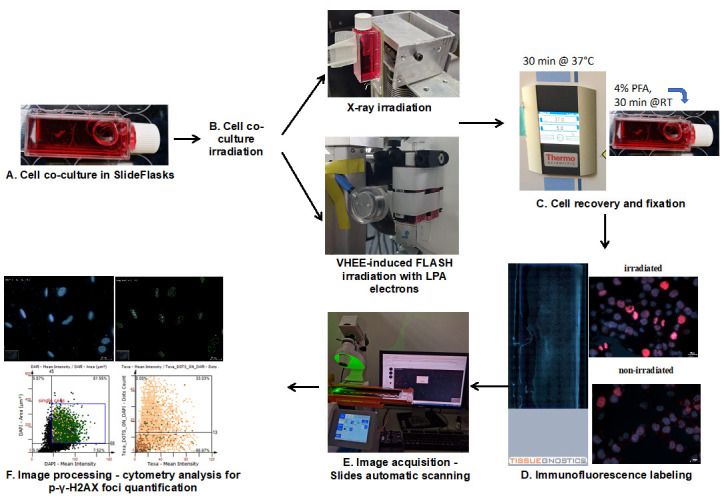


**Workflow of cell co-culture irradiation and foci quantification analysis**

## Background

Very high energy electrons (VHEE), defined as electrons with energies between 150 and 250 MeV, were proposed as an alternative radiotherapy approach and found adequate to penetrate deep tumor sites [1]. High-intensity laser-driven acceleration of VHEE of dose rates as high as 10^13^ Gy/s is achievable by laser-plasma accelerators (LPA) with quasi-monoenergetic beams, being proposed as new platforms for VHEE-induced FLASH irradiation [2,3]. We have recently confirmed the cell DNA damage–producing capacity of the laser-driven high-energy dose electrons produced by a tabletop high-power LPA in cancer cells for future FLASH-radiotherapy applications [4].

In order to investigate the potential sparing effect of FLASH radiation on normal cells while evaluating the conditions where it induces enhanced DNA damage to cancer cells, we set up a protocol that allows simultaneous exposure of normal and malignant cells to irradiation by LPA-produced electrons for comparison. The golden standard method in the field of radiobiology to evaluate the effects of radiation on cells is by analyzing nuclear foci that accumulate at DNA damage sites [5,6]. The ionizing radiation is known to produce DNA double-strand breaks (DSBs) that affect preponderantly the cells engaged in the cell cycle [7]. Therefore, this type of radiation is used in radiotherapy to eliminate highly proliferative cancer cells from tumors [8]. Upon exposure of cells to radiation or other DNA-damaging agents, the DNA repair molecular mechanism is activated [7]. Proteins accumulating and forming foci at the sites of insult can be used as markers to quantitate the extent of the produced damage [6], as well as for monitoring the kinetics of DNA repair [9]. Ideally, the radiation regimen should have a maximal impact on cancer cells without affecting the normal function of other cells in the tissue. Using our proposed co-culture protocol, one can simultaneously observe the effects produced by a certain radiation dose and regimen on other cells in the target tissue alongside the cancer cells. This approach has several key advantages, namely 1) minimizing experiment-to-experiment variability when comparing the effect of a certain irradiation regime (type of source and dose) on different cell types; 2) allowing cell–cell communication between different cell types that modulate the outcome on each cell type, similarly to events produced within the tumor microenvironment; and 3) minimizing the time required for radiation exposure, thereby increasing feasibility of large experiments where several doses have to be applied in replicates in the same day on cells from the same batch. Our method allows discriminating between different cell types within the same sample based on morphological and fluorescence parameters (image cytometry). Here, we present a case where different cell types have different nuclear shapes [A375 cells have round nuclei, while normal human epidermal melanocytes (NHEMs) have elongated nuclei]. Nevertheless, TissueQuest enables users to separate cell populations based on protein markers’ expression or other features that can be quantified using fluorescent labeling.

For this, we have harnessed the power of quantitative imaging that allows precise evaluation of foci number with increased throughput on a statistically significant number of cells per sample. Using an automatic scanning system and image cytometry software, we have recently compared the effects of VHEE radiation and conventional X-ray in co-cultures of melanoma (A375 cells) and NHEMs [4]. Upon recovery and labeling of irradiated cells with antibodies against p-γ-H2AX and a nuclear stain (i.e., Hoechst), we have successfully shown that the accelerated VHEE can induce an increase in DNA damage foci count in cancer cells. Noteworthy, in a specific area of the co-culture monolayer, the effect is only observed in cancer cells, while spearing the NHEMs from stress [4].

The protocol presented here can be adopted by researchers studying the effect of any type of radiation on adherent cells, as well as by investigators who apply other types of DNA damage agents to cells in vitro (e.g., cisplatin). Also, the detailed presentation of the quantitative cytometry analysis strategy will be useful for TissueFAXS microscopy users to implement automatic scanning and analysis in their laboratories.

## Materials and reagents

**Biological materials**

1. A375 human metastatic melanoma cells [American Type Culture Collection (ATCC), catalog number: CRL-1619)]. The cells should be grown to 70%–80% confluency in A375 culture media (see Recipe 1) and kept in a humidified environment at 37 °C, in the presence of 5% CO_2_, in a cell culture incubator

2. Normal human epidermal melanocytes (NHEMs) (Lonza, catalog number: CC-2504)

**Reagents**

1. High-glucose (HG) Dulbecco’s modified Eagle’s medium (DMEM) containing L-GlutaMAX (Gibco, catalog number: 61965-026)

2. Melanocyte growth basal medium 4 (MBM-4) (Lonza, catalog number: CC-3250)

3. Melanocyte growth medium 4 (MGM-4), Single Quots Supplements (Lonza, catalog number: CC-4435)

4. Fetal bovine serum (FBS) (Gibco, catalog number: 10437-028)

5. Penicillin-Streptomycin, 10,000 U/mL to 10,000 μg/mL (Gibco, catalog number: 15140122)

6. 1× Dulbecco's phosphate-buffered saline (DPBS) without calcium (Ca) and magnesium (Mg) (Gibco, catalog number: 14190250)

7. 0.05% Trypsin-EDTA solution (Gibco, catalog number: 25300054)

8. 0.025% Trypsin-EDTA solution (Lonza, catalog number: CC-5012)

9. 0.02% Versene (EDTA) solution (Lonza, catalog number: 17-711E)

10. Trypsin neutralizing solution (TNS) (Lonza, catalog number: CC-5002)

11. 0.4% Trypan Blue solution (StemCell Technologies, catalog number: 7050)

12. Bovine serum albumin (BSA) powder (Sigma-Aldrich, catalog number: A9647-10G)

13. Cisplatin powder (Santa Cruz, catalog number: sc-200896)

14. Mouse monoclonal antibodies for the Ser140 phosphorylated form of γ-H2AX (Invitrogen, Thermo, catalog number: MA1-2022)

15. Alexa Fluor 594-conjugated donkey anti-mouse secondary antibodies (Invitrogen, Thermo, catalog number: A21203)

16. Triton X-100 solution (Sigma-Aldrich, catalog number: T9284-500ML)

17. Paraformaldehyde (PFA) powder (Sigma-Aldrich, catalog number: P6148-500G)

18. Hoechst 33342 (Molecular Probes, Thermo, catalog number: H21492)

19. FluorSave Reagent (Millipore, catalog number: 345789)

20. NaOH (ADRA CHIM SRL, catalog number: 011-002-00-6)

**Solutions**

1. A375 cell culture media (complete DMEM HG) (see Recipes)

2. NHEM cell culture media (complete MBM-4) (see Recipes)

3. 10 mM cisplatin stock solution (see Recipes)

4. 5 M NaOH solution (see Recipes)

5. 4% PFA solution (see Recipes)

6. 0.2% Triton X-100 permeabilization solution (see Recipes)

7. 0.5% BSA blocking solution (see Recipes)

8. Primary antibody incubation solution (see Recipes)

9. Secondary antibody incubation solution (see Recipes)

10. Hoechst staining solution (see Recipes)

**Recipes**

**1. A375 cell culture media**

**Table:** 

Reagent	Final concentration	Quantity or Volume
DMEM HG medium	n/a	445 mL
Heat-inactivated FBS	10%	50 mL (see note*)
Penicillin-streptomycin solution	100 units/mL penicillin, 100 μg/mL streptomycin	5 mL
Total (optional)	n/a	500 mL

*Note: Before preparing the media, inactivate the bottle of FBS into a water bath for 30 min at 56 °C. Allow the heat-inactivated FBS (hiFBS) solution to cool down before adding it to the complete DMEM HG media. Prepare hiFBS aliquots and store them in the -25 °C freezer.

**2. NHEM cell culture media**

**Table:** 

Reagent	Final concentration	Quantity or Volume
MBM-4 basal medium	n/a	500 mL
MGM-4 Single Quots Supplements	n/a	9 mL
Total (optional)	n/a	509 mL

*Note: After adding MGM-4 Single Quots Supplements to MBM-4 basal medium, use within one month. Do not re-freeze.

**3. 10 mM cisplatin stock solution**

**Table:** 

Reagent	Final concentration	Quantity or Volume
Cisplatin powder	n/a	30 mg
MilliQ ultrapure water	n/a	Adjust to 10 mL
Total (optional)	10 mM	10 mL

**4. 5 M NaOH solution**

**Table:** 

Reagent	Final concentration	Quantity or Volume
NaOH pellets	n/a	20 g
MilliQ ultrapure water	n/a	Adjust to 100 mL
Total (optional)	5 M	100 mL

**5. 4% PFA solution**

**Table:** 

Reagent	Final concentration	Quantity or Volume
PFA powder	4%	2 g
PBS (see note *)	1×	Adjust to 50 mL
NaOH solution	n/a	3 μL sequential pipetting/dropwise until a clear solution is formed (~pH 7)
Total (optional)	n/a	50 mL

*Note: Heat 40 mL of PBS in a glass beaker under a fume hood to ~60 °C. Add the PFA powder and stir until it dissolves. Cool the solution to room temperature (RT) and then adjust the pH to 7 using NaOH. Bring the volume to 50 mL. Aliquot and store in the freezer.

**6. 0.2% Triton X-100 permeabilization solution**

**Table:** 

Reagent	Final concentration	Quantity or Volume
Triton X-100 solution (see note *)	as produced (0.2–0.9 mM)	100 μL
PBS solution	1×	50 mL
Total (optional)	0.2%	50 mL

*Note: Triton X-100 is a viscous solution. Cut the pipette tip before aspirating the solution. Release the tip in the PBS solution after resuspending it three times.

**7. 0.5% BSA blocking solution**

**Table:** 

Reagent	Final concentration	Quantity or Volume
BSA powder	n/a	0.25 g
PBS solution (see note *)	1×	Adjust to 50 mL
Total (optional)	0.5%	50 mL

*Note: Add small amounts of PBS solution onto the BSA powder and mix gently. Otherwise, the mixed solution will become foamy. If so, centrifuge at the maximum speed rotation allowed for the centrifuge tube in which you dissolved the powder.

**8. Primary antibody incubation solution**

**Table:** 

Reagent	Final concentration	Quantity or Volume
Mouse anti-p-γ-H2AX	1 mg/mL	
BSA blocking solution	0.5%	
Total (optional)	1:200	

**9. Secondary antibody incubation solution**

**Table:** 

Reagent	Final concentration	Quantity or Volume
AlexaFluor 594-conjugated donkey anti-mouse secondary antibody	2 mg/mL	
BSA blocking solution	0.5%	
Total (optional)	1:400	

**10. Hoechst staining solution**

**Table:** 

Reagent	Final concentration	Quantity or Volume
Hoechst 33342 stock solution	10 mg/mL	1 μL
PBS solution	1×	3 mL
Total (optional)	1:3,000	3 mL

**Laboratory supplies**

1. T-75 cell culture flasks (Corning Life Sciences, catalog number: 430641U)

2. T-25 cell culture flasks (Corning Life Sciences, catalog number: 430639)

3. SlideFlasks (Thermo Scientific, catalog number: 734-2107); the package contains a chamber detachment tool

4. Sterile Corning 24-well polystyrene tissue culture plates (Corning Life Sciences, catalog number: 3526)

5. Sterile 15 mL polypropylene centrifuge tubes (Corning Life Sciences, catalog number: 430790)

6. Sterile 50 mL polypropylene centrifuge tubes (Corning Life Sciences, catalog number: 430828)

7. 2 mL serological pipettes (Corning Life Sciences, catalog number: 4486)

8. 5 mL serological pipettes (Corning Life Sciences, catalog number: 4487)

9. 10 mL serological pipettes (Corning Life Sciences, catalog number: 4488)

10. Microcentrifuge tubes (ratiolab, catalog number: 5616027)

11. 12 mm diameter coverslips (CS), circular (Marienfeld, catalog number: 0111520)

12. Microscope slides (Marienfeld, catalog number: 1000200)

13. Forceps (Millipore, catalog number: XX6200006P)

14. Syringe needles

15. Pipette tips (compatible with your automatic pipettes)

16. Microscope cover glasses for chamber slides (Nunc/Thermo, catalog number: 171080)

17. Marienfeld counting chambers (Neubauer, catalog number: MARI0640130)

18. Microscope cover glasses 22 × 22 mm for counting chamber (Paul Marienfeld GmbH & Co, Marienfeld SUPERIOR, catalog number: 0101050)

19. Carl Zeiss Immersol immersion oil for microscopy (Zeiss, catalog number: 518F)

## Equipment

1. Cell culture incubator maintained at 37 °C and 5% CO_2_ (Thermo Scientific, model: Heracell VIOS 250i, catalog number: 13-998-253)

2. Laminar-flow biosafety cabinet (Thermo Scientific, model: KS 15, Class II)

3. Refrigerated centrifuge (Beckman Coulter, model: Allegra X-12R)

4. MilliQ water purification system (Millipore, Model SAS 67120 water purification with filter Biopak Polisher, catalog number: CDUFBI0A1)

5. Stripettor Ultra Pipet Controller (Corning, catalog number: 15536304)

6. Eppendorf Research plus P10 mechanical pipette (Eppendorf, catalog number: 3123000020)

7. Eppendorf Research plus P20 mechanical pipette (Eppendorf, catalog number: 3123000039)

8. Eppendorf Research plus P200 mechanical pipette (Eppendorf, catalog number: 3124000083

9. Eppendorf Research plus P1000 mechanical pipette (Eppendorf, catalog number: 3123000063)

10. X-ray system consisting of a NIKON XT H 225kV reflection target source with 3 μm focal spot size (Nikon Metrology NV) and a PTW UNIDOS T 10005-50406 Electromer connected to a Farmer ionization chamber TN30010 (calibrated at PTB Germany)

11. X-ray detector system: Gafchromic EBT3-810 dosimetry film (Ashland Global Holdings Inc., product code: 828204)

12. Customized chopper manufactured by selective laser melting (SLM) using 3D printing from steel with brass infusion. X-ray chopper specifications: 12 slits of 8 mm (height) × 1.5 mm (width) at 15° apart from each other

13. LPA system (Thales, France) based on a Ti:Sapphire high-intensity laser (800 nm, 25 fs pulse duration, 25 J energy at 0.1 Hz, 1 J at 10 Hz), beam transport (Ardop, France), interaction chamber (Astra, Gemini, RAL UK)

14. Electron beam detector systems: ionization chamber (model Advance Markus, type TN34045), three thermoluminescent dosimeters (model Panasonic), Gafchromic film (model GF-EBT3)

15. Automated imaging system (TissueGnostics, Vienna, Austria, model: TissueFAXSiPlus i12), based on the Zeiss Axio Observer.Z1 motorized inverted microscope appended with an ultra-precise motorized stage for automated sample acquisition with:

a. Oil immersion objective lens (Zeiss, model: Plan-Apochromat 63×/1.4 Oil DIC M27, 0.19 mm working distance)

b. Air objective lens (Zeiss, model: LD Plan-Neofluar 10×/0.3 M27, 5.3 mm working distance)

c. Air objective lens (Zeiss, model: LD Plan-Neofluar 20×/0.4 Korr M27, 7.9 mm working distance)

d. DAPI filter (excitation λ: 360 nm; emission λ: 462 nm), associated to the DAPI channel

e. Alexa 568/Cy3 filter (excitation λ: 568 nm; emission λ: 603 nm), associated to the TxRed channel

f. High-sensitivity digital monochrome camera for fluorescence microscopy (PCO AG, Kelheim, Germany; model: PCO PixelFly 14-bit dynamic range grayscale CCD camera; catalog number: C11440-10C)

g. Microscope fluorescence light source: Excelitas X-Cite 120PC Q (Cambridge Scientific, catalog number: 11419)

*Note: Full instrument configuration is available at* *https://www.biochim.ro/facility-11/* .

*Note: Due to the necessity of recovering the cells in a CO_2_ incubator before fixation (and also keeping them in a physiological milieu immediately prior to the electrons exposure), the irradiation facility should be close to a tissue culture lab. Alternatively, for short periods of time (up to 4 h), cells could be kept at 37 °C in HEPES buffered media. This should be tested before the experiment because not all cell lines cope well in these conditions.*

## Software and datasets

1. TissueFAXS Slides Module (version 3.5.5.0129, release date 20 August 2012)

2. TissueQuest (version 4.0.1.0140, release date 18 November 2014)

3. Microsoft Office 365 Excel

4. GraphPad Prism (version 9.5.1 (528), release date 24 January 2023)

## Procedure

**A. Setup of cell culture conditions for irradiation**

1. Culture vessels priming before cell seeding for the irradiation experiment: One day before the experiment, incubate each SlideFlask that will be irradiated or kept as non-irradiated control with a mix of 1 mL of A375 cell culture media and 1 mL of NHEM cell culture media. Keep them for 30 min in a humidified environment at 37 °C with 5% CO_2_ in the cell culture incubator before plating the cells.

***Note:*** *In parallel, it is advisable to seed similar samples in wells of a 24-well plate to be treated with drugs (e.g., cisplatin at IC50 or other DNA damage-inducing concentration) that induce the desired effect, as chemical positive controls. Make sure the surface quality of the wells is similar to the one of the irradiated vessels so they can be compared: borosilicate cover glass, tissue culture plastic, glass bottom chambered slide, etc. Cells have different attachment behaviors on different surface qualities with consequences on proliferation, confluency generation, and overall functionality.*

**Critical:** A coverslip dedicated to the secondary antibody-only negative control should be prepared for checking antibody specificity and setting the background threshold.

***Note:*** *Wait until the cell monolayers from your cell culture flasks are at approximately 80% confluency. Then, proceed with detaching and plating them in SlideFlasks for the irradiation experiment.*

***Note:*** *Prior to the experimental procedure, the number of cells to be seeded should be determined in order to obtain 80%–90% confluency the next day. Monolayers should not be overconfluent at the final endpoint, because they could easily detach from the surface during the washing procedures or even die and float before fixation.*

2. Cell seeding for the irradiation experiment: Culture the cells in SlideFlasks 24 h prior to the irradiation procedure. After growing your cells in specific culture vessels (e.g., T-75 cells culture flasks for A375 and T-25 flasks for NHEM cells) in the cell incubator, bring them to the laminar flow hood and proceed with cell detachment.

a. Aspirate the cell culture media from the T-75 cell culture flasks in which A375 were cultivated. Wash the cells using 10 mL of PBS. Detach the cells with 1 mL of 0.05% Trypsin-EDTA solution for 5 min in the cell incubator. Stop the trypsinization by adding 4 mL of culture media. Pipette up and down three times and transfer the 6 mL of cell suspension to sterile 15 mL centrifuge tubes. Centrifuge the cells at 200–300× *g* (1,200–1,500 rpm) for 5 min at room temperature (RT). Remove the supernatant and add 2 mL of culture media onto the cell pellet. Resuspend three times and harvest 500 μL of cell suspension to dilute it with 0.4% Trypan Blue solution for counting with Marienfeld cell counting chambers. Use a density of 840,000 A375 cells in 1 mL of A375 cell culture media to seed each SlideFlask for co-culture with NHEM cells.

***Note:*** *Dilute the cell suspension with the appropriate dilution factor of 0.4% Trypan Blue solution (i.e., 1:2, 1:5, 1:10) to count between 20 and 50 cells per each counting chamber 4 × 4 square field.*

b. Aspirate the cell culture media from the T-25 cell culture flasks in which NHEM cells were grown. Rinse the cells gently with 5 mL of PBS at RT by pipetting it down the cell-free surface of the culture vessel. Aspirate the PBS from the flask. Add 1 mL of 0.025% Trypsin-EDTA solution diluted with 1 mL of 0.02% Versene and incubate the cells for 1 min in the cell incubator. Neutralize with 2 mL of TNS at RT and wash the flask with 2 mL of culture media. Collect all the cells and transfer them to sterile 15 mL centrifuge tubes. Centrifuge the cells at 100× *g* for 3 min at RT. Throw the suspension and resuspend the cell pellet with 1 mL of culture media. Dilute with 0.4% Trypan Blue solution to count the cells. Use 420,000 cells in 1 mL of NHEM cell culture media for each SlideFlask co-cultured with A375 cells.

**Caution:** NHEM cells disassociate easily from the flask surface; work gently with them.

**B. Irradiation using LPA electrons or X-rays**

1. On the day of irradiation, fill up the SlideFlasks with media and seal them by completely screwing the caps. This is necessary to prevent cell drying when the flask is tilted and accommodated in the irradiation chamber.

***Note:*** *If irradiating co-cultures, use either a mix of equal volumes of complete media of the component cell lines or a standard complete media of a cell line that does not negatively affect the other ones present in the flask.*

**Caution:** If using vented caps, make sure the filter stays dry throughout the procedure so as not to encourage microbial infection, especially if the endpoint analysis timepoint is later than 16–18 h. Also, to prevent potential spilling of media through the vent, add parafilm around the cap.

**Figure 1.**
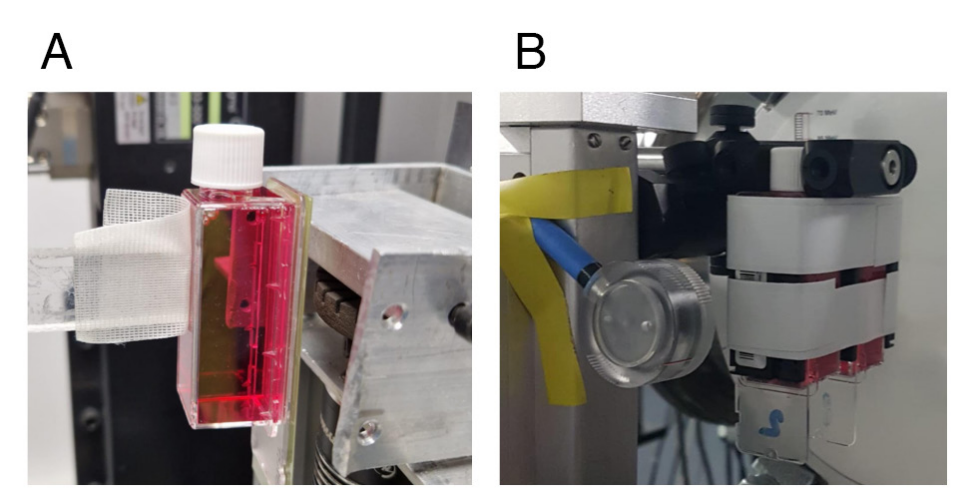
Photographic image exemplifying the mounting of media-filled SlideFlasks onto the support before targeting the sample with the X-ray beam (A) or LPA electrons (B)

2. For X-ray irradiation, mount the SlideFlasks vertically with double adhesive tape onto the vertical support placed in front of the beam-generating system (Figure 1A), making sure not to provoke vibrations or other mechanical stress that would detach the cells from the flask.

Pulsed X-ray setup: The setup used for delivering pulsed X-rays consists of an X-ray source and a customized X-ray beam chopper. It includes a sample holder that positions the sample in front of the head of the X-ray source. Operate the X-ray source at a voltage of 200 kV and a current of 100 μA using copper foil with a thickness of 0.2 mm as pre-filtration.

Chopper operation: Rotate the chopper at high speed using a brushless DC motor powered by an adjustable power source (24 V, 60 A) set at 19 V. Control the motor with an electronic speed controller unit. Regulate the rotational speed using an electronic board that generates a PWM (pulse width modulated) signal controlled by LabView using LINX library. Set the X-ray pulse frequency to 1.4 kHz.

Irradiation conditions: Position the sample at a distance of 10 cm from the X-ray source and irradiate with pulsed X-rays for 15 min and 30 s. To ensure optimal shielding of the X-ray beam passing through the chopper slits and to reduce the X-ray scattering, place Pb collimators in front of the X-ray source (aperture of ~4 mm, total thickness of ~0.8 cm). Carefully align the chopper slits with the collimator aperture and the center of the sample.

***Note:*** *For these operating parameters, under continuous exposure, a dose rate of 26 mGy/s at a distance of 10 cm was measured by an ionization chamber connected to a standard UNIDOS dosimeter.*

Dose measurements: Use Gafchromic EBT3 films to estimate the doses delivered to the samples. Place them on the back of the SlideFlask, in front of the plastic-attached cells, as viewed from the source. Prior to experiments on cell cultures, irradiate EBT3 films with precisely delivered doses, and generate calibration curves from the radiochromic film darkening, according to Campajola [14].

***Note:*** *In our experiments, the samples irradiated with pulsed X-rays for 15 min and 31 s received a total dose of 2.4 Gy, according to Gafchromic EBT3 measurements.*

3. LPA setup: The setup used for delivering very-high-energy electrons (VHEE) consists of a high-intensity laser beam focused with an off-axis parabolic mirror with a 3.2 m focal length into a supersonic gas jet consisting of 99% He + 1% N_2_. Use high-intensity petawatt laser-driven accelerators that can deliver very-high-energy electrons (VHEEs) at dose rates as high as 10^13^ Gy/s in very short pulses (10^-13^ s).

***Note:*** *For monitoring the effective applied dose, you can apply various radiation dose detectors and sensors on the flask surface close to the cell monolayer (e.g., in situ dose monitoring by ionization chamber, thermoluminescent dosimeters (TLDs) for evaluating cumulative dose post-experiment, or Gafchromic films for dose uniformity check).*

The quasi-monoenergetic electron energy distributions can be measured with a spectrometer equipped with a Pb collimator and magnetic dipole of 0.8 T, which can be set up into the electron beam at the extension of the interaction chamber.

As an example, for simultaneous irradiation of two containers (Figure 1B) with cells grown either in the first or both containers, introduce three TLDs: one placed in front of the first container, the second placed between the two containers, and the third placed after the second container. In our setup, the electron beam travels approximately 198 cm in vacuum before reaching the first TLD, and then it travels successively through a 60 μm Al foil, a 1 cm thick glass window, another 60 μm Al foil, a 0.5 mm cardboard, the scintillator screen LANEX, and 2 cm in air.

***Note:*** *In our experiments, the samples irradiated with VHEE received a total dose of approximately 150 mGy, according to ionization chamber dosimetry measurements.*

**General note:** Irradiate cancer cells and normal melanocyte co-cultures with either VHEE produced by LPA or pulsed X-ray beams (for control experiments). X-ray equivalent doses of up to 8 Gy are commonly used to study radiation response on in vitro cultured cell line models [15].

**C. Cell recovery and fixation**

1. Upon irradiation, allow cells to recover and accumulate phosphorylated γ-H2AX [10] at the double-strand DNA breaks for 30 min in the incubator.

***Note:*** *If flasks do not have vented caps, slowly release the cap to allow gas passage into the vessel. Remove a fraction of the media to prevent overflow; leave enough media to cover the cells and consult the working volume recommended by the vessel producer.*

2. Aspirate growth medium and gently wash cells once with a generous volume of PBS (at least equal to the media volume used for culture) at RT.

3. Fix cells using 4% PFA for 30 min at RT. Use a sufficient amount to cover the cell monolayer.

**Caution:** PFA is neurotoxic. Use a chemical hood or work in a well-ventilated space when performing the procedure. Also, all derived chemical hazardous waste should be collected separately and sent to appropriate facilities for neutralization. Do not discard solutions into the sink!

4. Aspirate fixing solution and gently wash cells twice with PBS at RT. Use at least double the amount of PFA used.

**Pause point:** Fixed cells can be kept in PBS at 4 °C for 1–2 weeks before immunofluorescent labeling. It is, however, advisable to proceed with the next steps immediately or within days as longer storage may reverse fixation affecting cell morphology and epitope integrity. Refill wells/flasks with PBS periodically and/or seal with parafilm to prevent cell drying.

**D. Immunofluorescence labeling**

1. Aspirate PBS from fixed co-culture cells plated on SlideFlasks or coverslip (CS) controls. Add a volume of 3 mL/SlideFlask and 350 μL/CS of 0.2% Triton X-100 permeabilization solution. Incubate for 3 min at RT.

2. Wash twice with PBS and incubate for 1 h at RT with 0.5% BSA blocking solution using the same volume as in the permeabilization step.

3. Before the immunostaining step, detach the flasks from the slides with the aid of the chamber detachment tool provided within the SlideFlasks package.

4. Add 350 μL/slide and 250 μL/CS of primary antibody incubation solution, respectively (except for the negative “secondary only” control, where you add blocking buffer). Place the slides in a horizontal position facing upward in a humidity chamber incubation box (any type of rectangular, transparent food box can be repurposed into a humid incubation chamber by placing wet napkins on its bottom to create a humid atmosphere; additionally, a plastic grid can be used to support the slides deposited onto the napkins and prevent slide wetting; one can also use a pipette tips box from which the tips’ support part is removed). Put the lid on the box and leave the primary antibodies to react for 30 min at RT.

***Note:*** *If using other antibody clones, please check the appropriate dilution factor and the incubation time recommended by the manufacturer in the product information sheet. If not mentioned, test them on your specific cell line.*

5. Rinse three times with PBS for 5 min each step.

6. Label the cells for 20 min with secondary antibody incubation solution: add 350 μL/slide and 250 μL/CS, respectively. Incubate the slides in a humidity chamber incubation box covered beneath with wet napkins to create a humid atmosphere, as in step 4.

**Critical:** Include here the secondary-only negative control for demonstrating signal specificity and setting the background threshold.

**Critical:** At this step, cover the box with aluminum foil to prevent light from damaging the fluorophores conjugated to the secondary antibodies.

7. After three washes with PBS, incubate the cells at RT for 1 min with Hoechst staining solution.

8. Rinse three times with PBS and once with MilliQ water to prevent the formation of salt crystals.

9. Use 100 μL/slide and 15 μL/CS of FluorSave Reagent, respectively, to mount the samples with the cell culture surface onto the microscope slides.

**Critical:** If you use circular 12 mm CSs to grow cells into 24-well plates, make sure the CSs mounted on a microscope slide are placed in the center and not at the distal areas of the slide, where the holder edge will mask the visualization of the samples. Make sure to leave the slide edges free when mounting the CS(s) to allow the slide to fit into the microscope’s slide holder.

**Pause point:** Mounted slides can be kept in the dark (covered with aluminum foil or stored in a dedicated slide box/cardboard folder) at 4 °C for up to one month before image acquisition without significant signal loss. It is however advisable to proceed with the next steps immediately or within days, as longer storage may favor the development of air bubbles due to drying of the mounting solution layer. To prevent this, one can seal the coverslips with transparent nail polish.

**E. Image acquisition: slides automatic scanning**

1. Start up the imaging system (power source, halogen lamp, microscope, PC).

2. Open the software that controls the imaging instrument by double-clicking the specific icon on the computer desktop. Make sure the slides’ sample holder is mounted on the stage and its planarity is optimal. Calibrate the motorized stage (if available) with the proper slide holder installed.

***Note:*** *In the case of the TissueFAXSiPlus system, open the TissueFAXS Slides module on the workstation desktop and calibrate the stage for slide acquisition as prompted by the software (see General note 1).*

3. Carefully fit the slides into the holder with the cell-covered surface toward the objective as for any inverted microscope visualization.

4. Start sample observation and image focusing using the objective with the smallest magnification available on your imaging instrument. The light path should be set to the *ocular* visualization mode, and the excitation lamp should be switched on. To easily find the correct focal plane, use the DAPI channel for cell nuclei visualization first. Then, check the signal on the TxRed channel to visualize phosphorylated γ-H2AX (p-γ-H2AX) that labels damaged DNA foci.

***Note:*** *Whenever you pause visualization of the samples, switch the lamp to standby mode to prevent photobleaching in the illuminated area of your sample.*

5. Prepare the system for image acquisition. For this, set up a new project using the image acquisition software. Give the new file a name and browse to select the directory for saving and storing the data.

***Note:*** *For TissueFAXSiPlus users, make sure the right image acquisition template is selected from the software menu before creating a new experiment project, by selecting Tools/Options/Default Experiment Settings/Preview. Choose slide orientation (e.g., Label Up/Label Down). One can choose to scan the whole slide, the center part of the specimen, or a custom region created by the user. Create a new project for a generic sample [not TMA (tissue microarray)] and select a 5× objective for preview and a 63× oil objective for field of view (FOV) acquisition. Select DAPI and TxRed channels for sample imaging. Save the file in the Setup folder by creating a subfolder with your name/project and a sub-subfolder with the name of the experiment.*

**Critical:** In the case of TissueFAXSiPlus system, make sure to define the desired location on the computer where the project file and associated image folders will be stored before sample acquisition. Also, do not perform any modifications to the data storage folders post-acquisition, such as moving the folders to a different location or renaming folders. The acquisition software (or the TissueFAXS Viewer visualization software) will not be able to retrieve the FOVs to generate stitched images of region overviews anymore!

6. Start live imaging through the camera by clicking the dedicated button in the visualization window on your computer screen. The light path should be set to camera visualization mode using a proper camera dedicated to fluorescence image acquisition.

***Note:*** *In the case of the TissueFAXSiPlus system, you should choose the PCO PixelFly camera attached on the right side of the microscope by selecting the right (R) side port in the TissueFAXS Slides software window dedicated to visualization mode. This action can also be performed by selecting the R side port from the light path specified on the Zeiss microscope digital display.*

***Note:*** *If the system has more than one available digital camera, make sure you select the correct camera from the imaging software menu. In the case of the TissueFAXSiPlus system, select the* camera manager *tab in the Tools/Options menu and choose PCO PixelFly from the dropdown list.*

7. **(Optional)** If you have an automated scanning system, you can perform *sample preview* to generate a sample map and define specific regions of interest (ROI) areas to be scanned. For this, use a low magnification objective (e.g., 5× objective) and the DAPI signal detection channel. Refine focus and use exposure parameters that allow nuclei visualization.

***Note:*** *For the TissueFAXSiPlus system, double-click the* Preview *tab in the top-left side of the software window and press* Acquire *after selecting the slide position specific to your sample (the holder may have 8 or 12 slide positions). Choose Current focus position when prompted by the dialog window.*

At the end of the scanning step, define the specific areas (ROIs) to be scanned using a high-magnification objective (e.g., 63× oil objective). For this, double-click the obtained preview to generate a separate enlarged visualization window. Here, choose on the left side the desired tool to define regions with a specific geometry (e.g., rectangle, circle, custom freeform). Use the mouse to select the ROI on the previewed slide. A region will be automatically created under the selected slide. Rename “Slide x” (x is the number defining the slide position into the holder) to the name of your sample. Rename “Region 001” to the name of your ROI. Repeat the creation of ROIs for all areas to be investigated.

**Critical:** In the case of the TissueFAXSiPlus system, make sure to define slide and region names before acquiring! No modification is possible after the acquisition.

8. Set the exposure times specific for each channel (e.g., DAPI or TxRed) and objective magnification (e.g., 63× oil objective) using both negative and most brightly stained controls to ensure that fluorescent signals are within the dynamic range of the markers’ expression. Use the prepared secondary-only sample to set the background threshold. For p-γ-H2AX signal detection setup, use the cisplatin (CisPt)-treated cells sample to fit the bright intensity signal into the visible dynamic range without detectable overexposure. Save the set parameters for the chosen magnification and do not perform any modification within a sample set to allow objective comparison between experimental conditions.

***Note:*** *In the case of the TissueFAXSiPlus system, you can start by pressing the Auto button for exposure time for each channel and refine exposure time (ideally no more than 300 ms exposure time) and background lower and upper thresholds to optimize signal-to-noise ratios.*

9. Start sample image acquisition. Use either an automating image scanning instrument (such as the TissueFAXSiPlus or any other slide scanning system) to sample representative regions (ROIs) of your samples or capture a sufficient number of images with a total number of cells >500 per sample (as in [11]) for quantitative analysis.

***Note:*** *In the case of the TissueFAXSiPlus system, go to Tools/Options and select the* Focus *tab in Scan Settings to select sample focusing mode (e.g., Autofocus/Manual/Current position). For the best fine-tuning, select Manual. For the most efficient scanning speed, select Autofocus and press “Set around this point” after manual optimization of focus using the microscope fine-tuning knob. Press Save and Exit to save and close the dialog window. Select the region to be acquired and right-click to open the associated drop-down menu; press Acquire to start scanning the selected region*.

**Caution:** If using Autofocus, make sure the sample does not contain artifacts; otherwise, the focus will be incorrectly diverted toward them. After the scanning has finished, check the quality of the images carefully and correct the FOVs with non-optimal focus. This can be done by selecting the flag icon above the ROI image and clicking the FOVs to be corrected. Then, right-click to select the desired re-acquisition mode (e.g., “reacquire FOV”). Images will be overwritten so that the selected FOVs can be corrected.

***Note:*** *Images of all samples within the experiment should be acquired on the same day if possible, without modifying any of the excitation or imaging parameters (lamp excitation level, exposure time, background threshold levels, room illumination, etc.) to ensure consistency. Ideally, the microscope should be placed in a dark room for fluorescent imaging; otherwise, use blinders to block sunlight passing through the windows. Sunlight affects fluorophores conjugated to secondary antibodies. Also, do not spend too much time on a specific region on the sample during the initial (exposure time) setup to prevent photobleaching. Any variation in acquisition conditions will produce errors in signal quantitation and sample comparative analysis.*

10. Repeat step E9 for all samples and regions to be analyzed.

**Figure 2.**
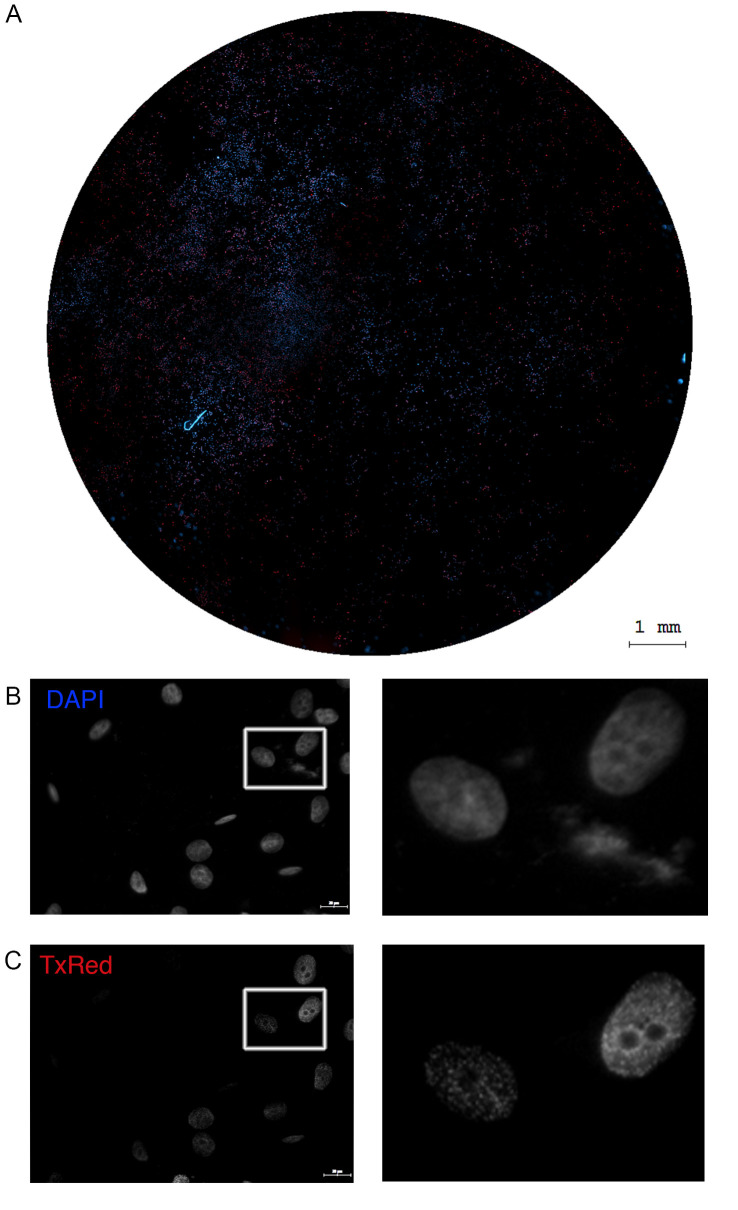
Example of an automatic image scanning result obtained using the 63× objective. A single .aqproj file consists of images captured on the DAPI and TxRed fluorescence channels for all samples and regions in a project. One region overview (A, scale bar: 1 mm) and one field of view (FOV) (B, scale bar: 20 μm) are shown for exemplification. Separate DAPI and TxRed channel images and enlarged insets are presented in grayscale (B). The p-γ-H2AX signal localizes to the nucleus in discrete regions known as foci.

***Note:*** *In the case of the TissueFAXSiPlus system, all images are automatically stored in the folder associated with the project as grayscale captures. For pseudocolor visualization, open the files using the acquisition software and export as .tiff or .jpeg files in color mode, as individual channel-specific images or as merged versions of the overlapped channels*. *One can export region overviews (Figure 2A) or specific FOVs (Figure 2B) using the* Export *button situated at the top of the generated scan*.

**Pause point:** Once images are saved and stored into folders, quantitative analysis can be performed anytime afterward.

**F. Image processing: cytometry analysis for p-γ-H2AX foci quantification**

**Figure 3.**
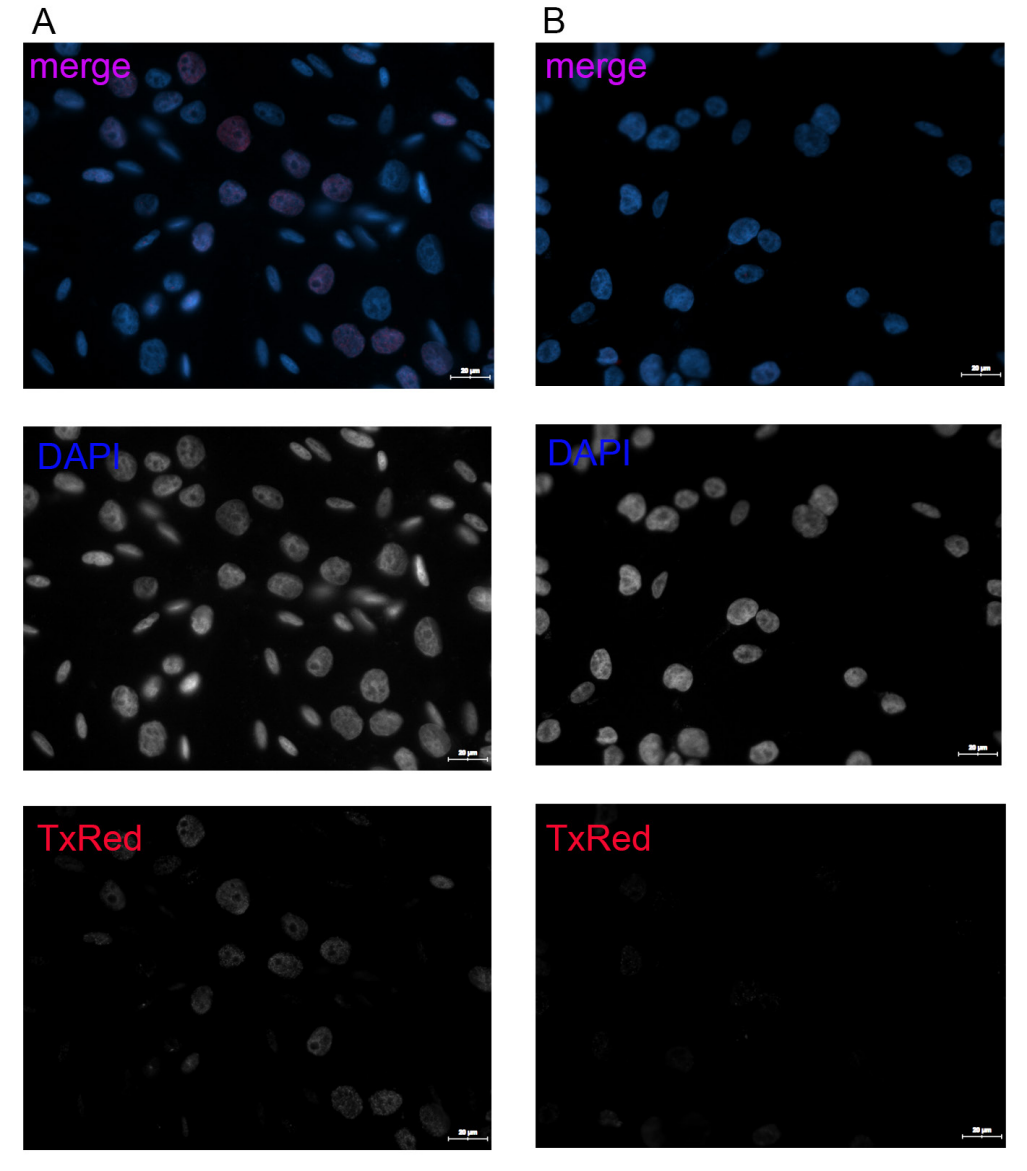
Example of images obtained from CisPt-treated cells and untreated controls using the 63× objective. Overlapped DAPI and TxRed FOV colored images, as well as separate grayscale DAPI and TxRed channel images, are presented. The merged images of both channels as well as the TxRed-captured images show that p-γ-H2AX signal is enhanced upon treatment with CisPt (A) as compared to the untreated cells (B). The small, elongated nuclei represent NHEM cells, while the circular bigger ones belong to A375 cells. Scale bar: 20 μm.

Images of the positive control sample should be used as a reference to set the parameters’ cutoffs and the gates for foci quantification, using the forward and backward gating functions of the software. In the case of Orobeti et al. [4], A375 cells that were treated with 2 μM CisPt for 24 h were used to set up parameters. Examples of images taken for DAPI and TxRed signals are shown in Figure 3.

1. Open the TissueQuest fluorescence image cytometry software by double-clicking the shortcut icon on the desktop.

***Note:*** *For this* Bio-protocol, *we specifically exemplify how to use the TissueQuest image cytometry software associated with the TissueFAXS scanning instruments to automatically count and analyze nuclear foci (see General note 1). However, analysis can also be performed with other image analysis packages such as the freely available ImageJ using a particle analysis plugin to quantify the foci with each image being processed separately [12]. Inversely, one can acquire separate images or region overviews with other fluorescence microscopes or automatic scanners and upload them into TissueQuest for quantitative analysis.*

**Figure 4.**
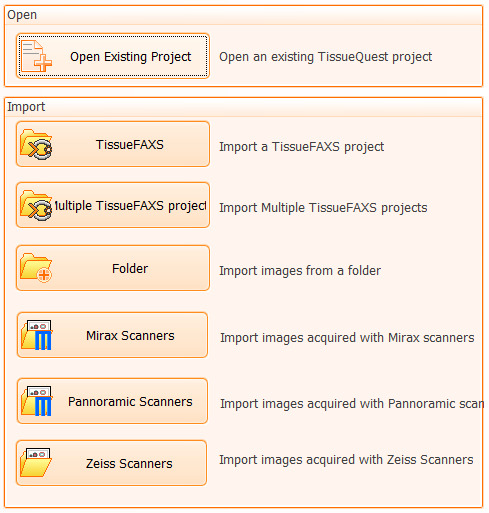
Screen capture of the “TissueQuest Open/Import” dialog window

2. In the opened dialog window (see Figure 4), select the option “Import a TissueFAXS project” and choose the .aqproj project file from the specific experiment folder. This will initiate ROI image data uploading into the analysis software.

***Note:*** *If you want to continue or modify an already initiated quantitative analysis, select the option “Open an existing TissueQuest project.” If you want to analyze samples imaged with other microscope systems, choose the appropriate selection corresponding to the instrument you used (e.g., Mirax Scanners, Panoramic Scanners, or Zeiss Scanners).*

**Figure 5.**
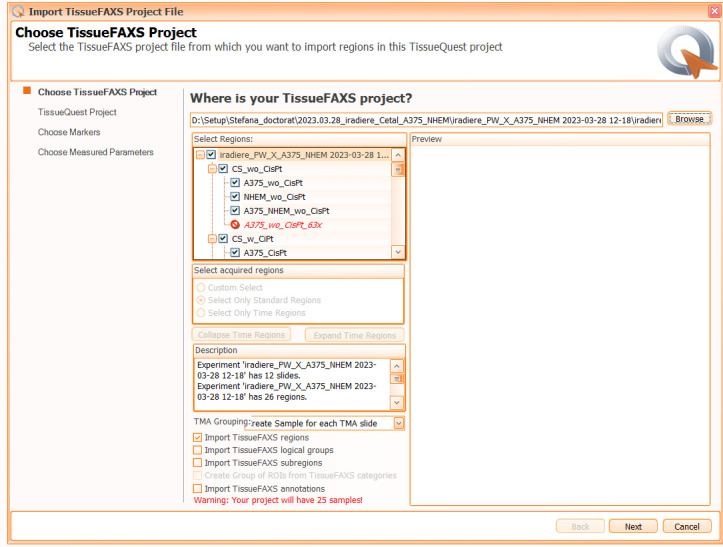
Screenshot of the “Choose TissueFAXS Project” dialog window

3. In the *Choose TissueFAXS Project* window (see Figure 5), select the slides and regions to be included in the analysis by checking the corresponding boxes and press *Next*.

***Note:*** *One can initiate a new TissueQuest analysis anytime with differently set parameters based on the same TissueFAXS acquisition; alternatively, one can append an already performed analysis with new selected regions from the same acquisition project and reapply the same analysis strategy onto those, for uniformity.*

**Figure 6.**
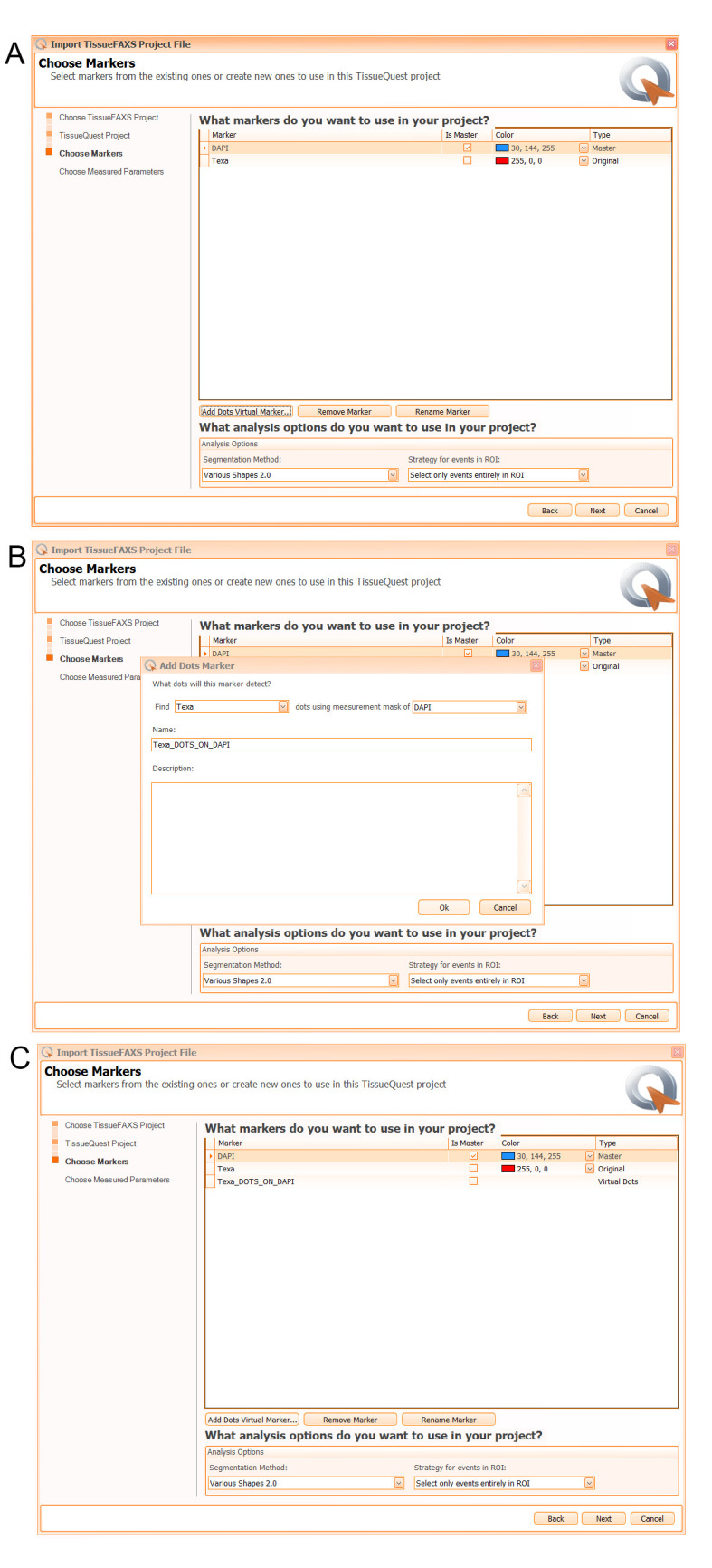
Snapshot of the *Choose Markers* dialog window and the *Add Dots Marker* option. A. DAPI and Texa markers are shown by default based on the information from the scanned project. B. One can add additional markers, such as a dots marker using a virtual marker. C. The Texa_DOTS_ON_DAPI marker is added to the markers list of the project to analyze Alexa Fluor 594 labeled nuclear foci that overlaps with the Hoechst stained nucleus detected on DAPI.

**Figure 7.**
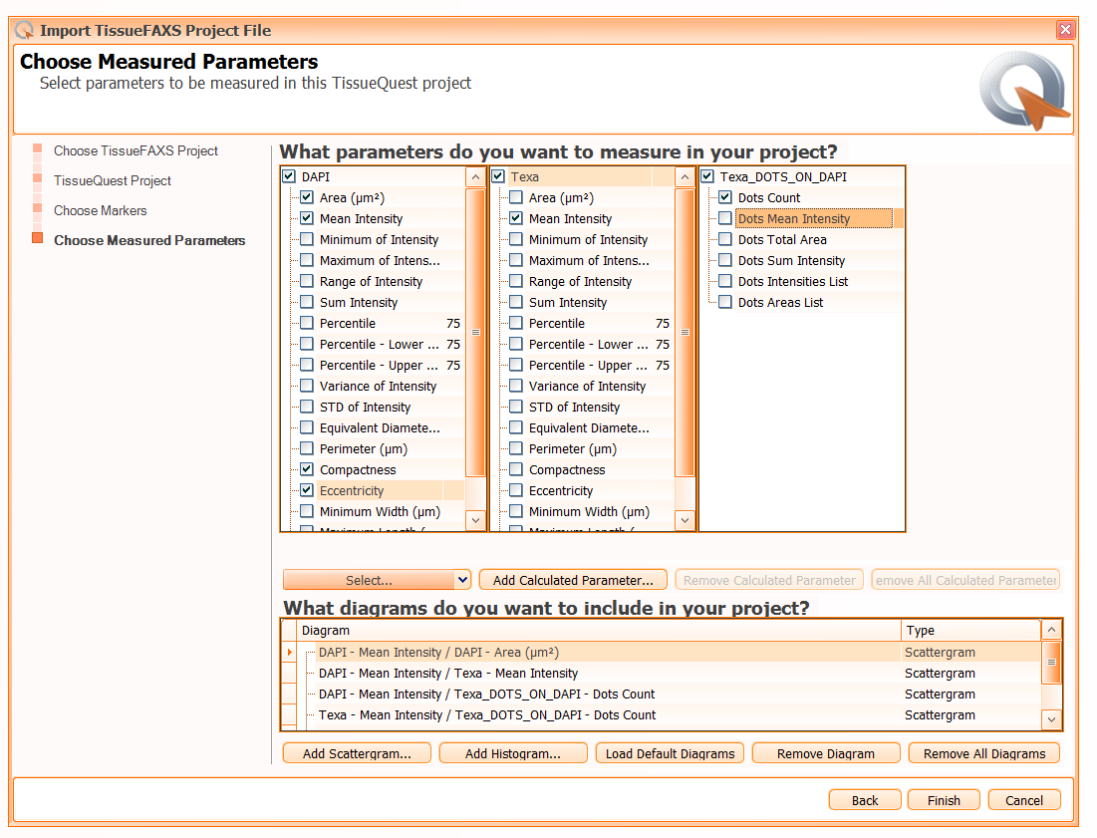
Screenshot of the *Choose Measured Parameters* dialog window

4. In the *TissueQuest Project* window, select the desired location to store the project analysis. By default, a project folder will be created in the same folder where the initial image acquisition project is stored. Optionally, you can add notes describing the experimental details. Click *Next*.

5. In the *Choose Markers* window (see Figure 6A), add/remove/rename the fluorescence markers (i.e., channels) for the desired analysis (e.g., DAPI and Texa). By default, DAPI is the master channel, as the single cells are identified based on the nucleus staining. You can change this if you use other nuclear dye.

6. Click on *Add Dots Virtual Marker* (see Figure 6B) to add a virtual channel on TxRed to define Texa_DOTS_ON_DAPI. Click *OK*.

7. Choose *Various Shapes 2.0* and *Select only events entirely in ROI* for analysis and press *Next* (see Figure 6C).

8. In the *Choose Measured Parameters* window (see Figure 7), select the parameters to be used (e.g., DAPI Area, DAPI Mean intensity, DAPI Compactness, Texa Mean Intensity) for quantitative analysis on each channel. Additionally, choose *Dots count* as a parameter on the created Texa_DOTS_ON_DAPI virtual channel.

***Note:*** *For co-cultures where cell lines can be distinguished based on nuclei elongation, one can use either the eccentricity parameter [4] or the Feret ratio on DAPI [13] (both defining nuclei circularity index based on the ratio between their major and minor axes).*

9. Add or remove specific scattergrams or histograms from the diagram list of the analysis project. Proceed to the analysis by clicking on the *Finish* button.

***Note:*** *The sequence of diagrams can also be changed at any step during analysis using the dedicated Diagrams action button.*

**Figure 8.**
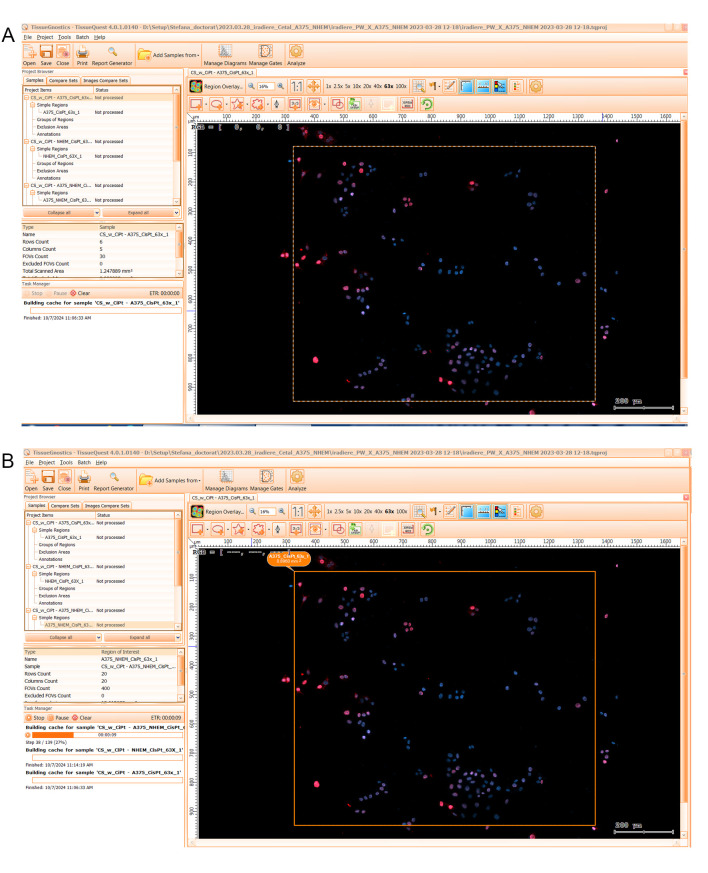
Screen capture of the TissueQuest analysis window (left screen) depicting menu tabs in the menu bar and a list of samples in the Project Browser mini-window. By default, an image of the first simple region in the sample list is open (A). Cache building progress is visible in the orange status bar and the date and time when the process is finished is shown at the end (B).

**Figure 9.**
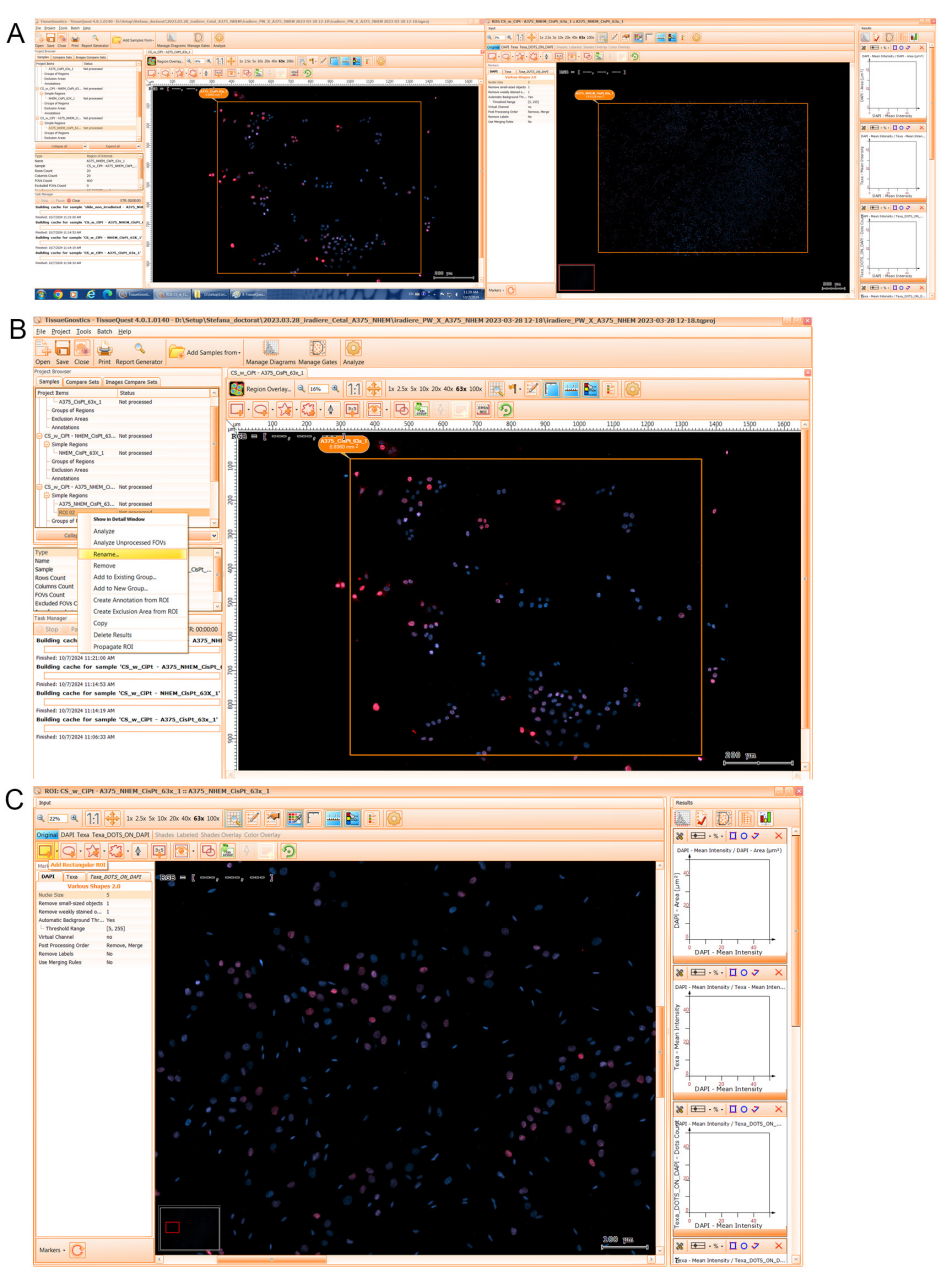
Screen captures of the software windows used to segment the nuclei and analyze the DAPI and TxRed signals. Key actionable buttons and tabs are visible. A. Left-side and right-side monitor views of the TissueQuest software. B. Creation of a setup ROI within the positive control sample. C. Fine-tuning of marker pixel intensity thresholds for image segmentation.

10. When the analysis window opens (see Figure 8A), click each region one by one and wait for the cache to be built for all of them. Progress is visible by the filling of an orange status bar (see Figure 8B).

11. Select the region drawn onto the positive control sample (i.e., cells treated with CisPt to induce DNA damage response) from the sample list to define an area for positive signal threshold setup. Double-click on it to open the region in a separate *Input* window on the right-hand screen (see Figure 9A). The tab corresponding to the *original* image (i.e., merged channels color view) visualization mode is selected. Zoom in and use the rectangle *Add Rectangular ROI* tool to enclose a representative cell subpopulation with various levels of signals on the TxRed channel including the dimmest and the brightest foci, and assign it a name (e.g., “Set-up region”) in the sample list (see Figure 9B). Choose this sub-region when prompted by the software to use it for fine-tuning the signal thresholds for each parameter; it will appear on the right-side screen (see Figure 9C).

***Note:*** *If one used the entire sample region for analysis setup, it would take a considerable amount of time for each iteration step in the algorithm.*

**Figure 10.**
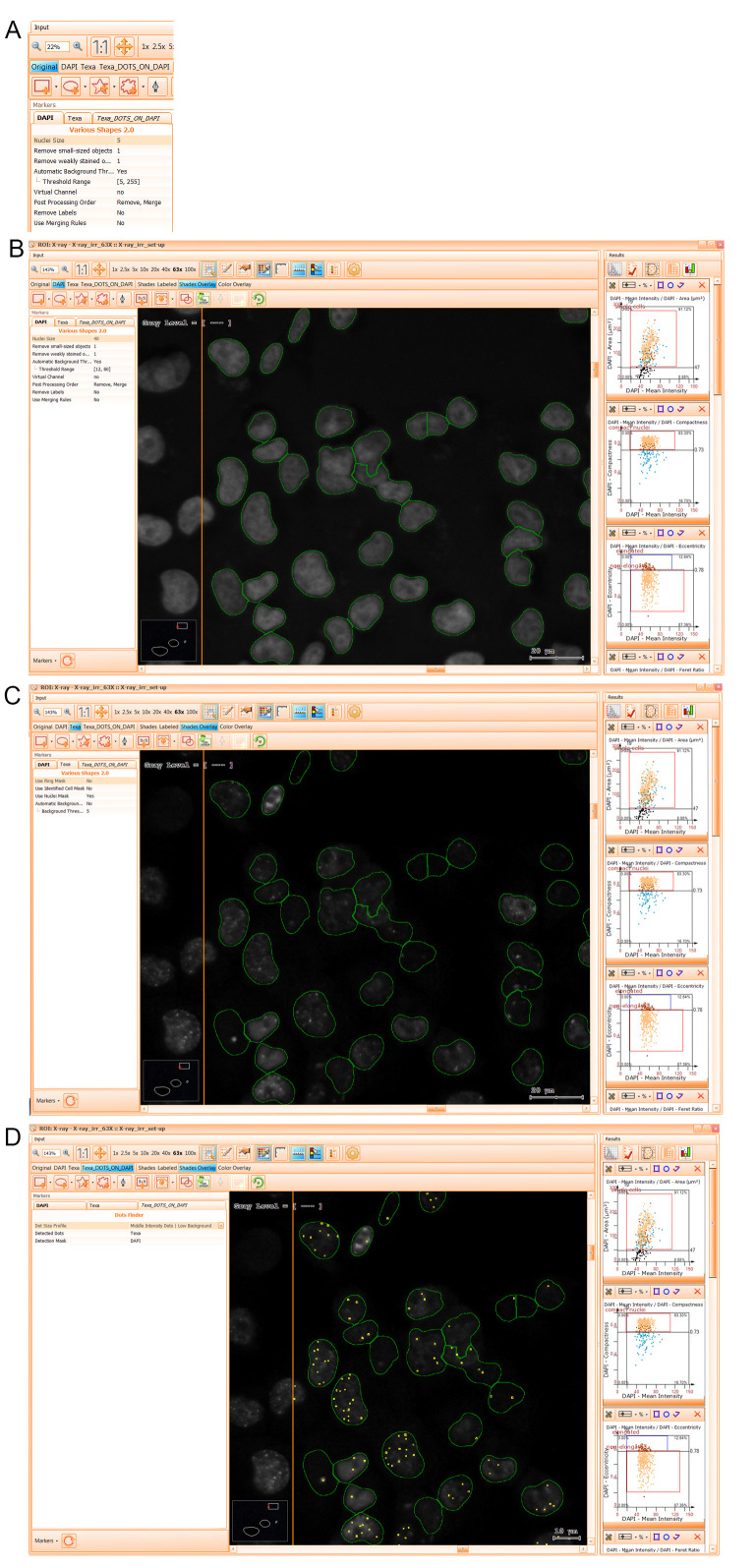
Obtaining segmentation masks. A. Magnified view of markers analysis criteria input panel. B. Example of a result obtained from the nuclei segmentation. C. Example of a result obtained from the Texa nuclear signal segmentation for p-γ-H2AX. D. Example of a result obtained from the Texa_DOTS_ON_DAPI nuclear p-γ-H2AX foci signal segmentation.

12. To identify single cells, first choose in the right-side screen *Input* window (see Figure 10A) the tabs corresponding to the *DAPI channel* to visualize nuclei in grayscale mode and *Shades Overlay* to observe the segmentation result in green. Then, fine-tune algorithm parameters in the *DAPI* tab of the *Markers* panel (see Figure 10B) and press the *Analyze* button marked with the *Settings* symbol above in the *Input* window. Do as many iterations as needed until the nuclei are correctly segmented (i.e., the encircled green masks overlap optimally with the visible DAPI-positive nuclei).

***Note:*** *We found the nuclei size of 40 (a.u.) and the DAPI threshold range of 12–80 to be optimal for our cells (see Figure 10B).*

***Note:*** *Dots corresponding to individual cells analyzed will appear in the scattergrams. Their projection on the axes corresponds to the parameter value for the specific cell.*

13. To identify the TxRed signal, choose “Texa” from the *Input* tab and from the *Markers* tab. Here, select “Yes” for “Use Nuclei Mask” in order to use the generated nuclei mask on DAPI as the location for Texa signal quantification (see Figure 10C). Then, fine-tune background threshold parameters to optimize signal detection.

***Note:*** *We found a background threshold of 5 to be optimal for our TxRed signal-to-noise ratio (see Figure 10C).*

14. To identify the foci, first choose the Tab corresponding to “Texa DOTS_ON_DAPI” virtual channel to visualize nuclear foci encircled in yellow. Then, fine-tune algorithm parameters in the *Texa* tab of the *Markers* panel (see Figure 10D) and press the *Analyze* button. Do as many iterations as needed until the p-γ-H2AX foci are correctly segmented (i.e., the encircled yellow regions overlap optimally with the visible foci).

15. After the segmentation is done, set up the cutoff thresholds for each parameter analyzed (i.e., DAPI Area, DAPI Mean intensity, DAPI Compactness, DAPI Eccentricity, Texa Mean Intensity, Texa DOTS_ON_DAPI). First, make sure that all necessary scattergrams (2-parameter diagrams) and histograms (1-parameter diagrams) are visible. To add or remove elements, select the *Manage Diagrams* icon in the upper right *Results* menu above the scattergrams and histograms series and select the ones needed from the list. Press *Close*. To set up cell population thresholds, click on the *Set Cutoff* button in the upper left corner of each diagram and apply it with the left click of the mouse in the desired position.

**Figure 11.**
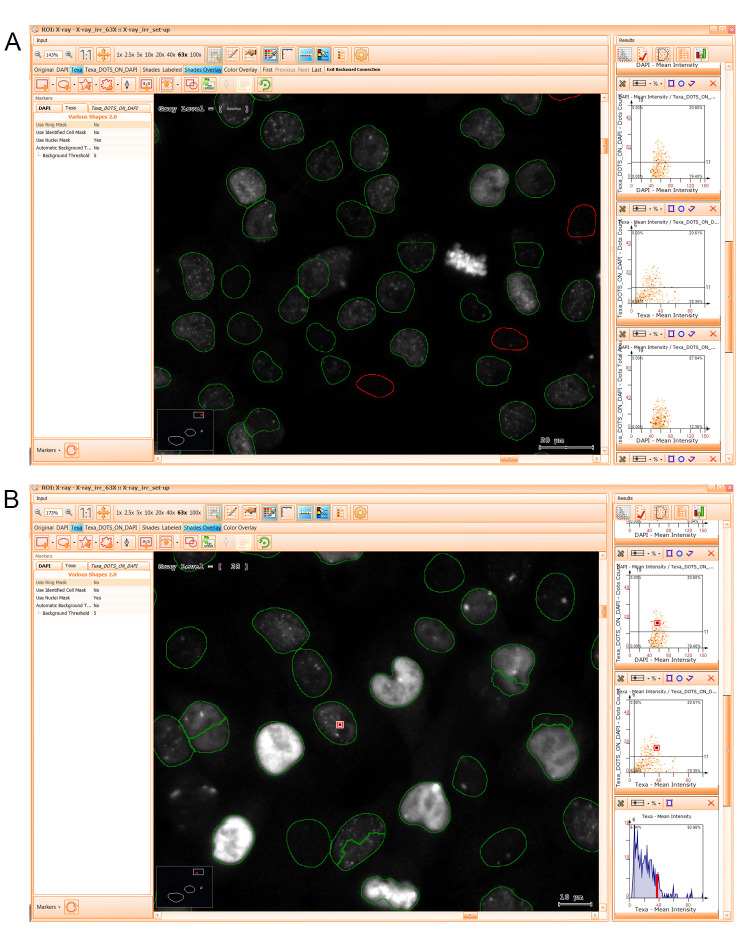
Backward and forward gating tools. A. Use of backward gating to visualize the impact of choosing a certain cutoff value. B. Use of forward gating to visualize the signal intensity for a certain cell.

**Critical:** Use forward and backward gating after each refining step to check if the negative and positive events (i.e., cells) are properly identified (see Figure 11). Right-click on the selected scattergram and select *View Backward Data For Left Quadrants* to visualize in red the events negative for the parameter detected on the horizontal axis (see Figure 11A for an example; Texa negative events in the second scattergram are shown in red). Right-click on the selected scattergram and select *View Backward Data For Lower Quadrants* to visualize in red the events negative for the parameter detected on the vertical axis. Right-click on the selected histogram and select *View Backward Data For Left Quadrants* to visualize in red the events negative for the parameter detected on the horizontal axis. Alternatively, the detected positive events can also be visualized by backward gating. Forward gating can be used by double-clicking to select a cell and check its quadrant location (see Figure 11B). After each step, check if false negative or false positive events are erroneously identified. If this is the case, adjust cutoff values by pulling it with the mouse toward the correct direction to include all positive events in the analysis and exclude all negative ones. Do this for all diagrams in the series.

16. After the cell population cutoffs are set up, initiate the gating sequence. For this, select each scattergram in the series and generate gates to define the cell population to be analyzed and filter out all unwanted events. To draw gates, press one of the three gate shape icons depicted in blue for the specific scattergram (rectangle/circle/freeform), define the events population by left-clicking, and release it after you enclose the cell subset. You can readjust by pulling the shape sides. Gate the intact cells by selecting the upper-right quadrant of the DAPI Area/DAPI Mean Intensity scattergram. Go to the *Manage Gates* button and assign a name and color (e.g., blue) to gate the “single cells.” Then, go to the DAPI Compactness/DAPI Mean Intensity scattergram and select the previously gated events by choosing the “single cells” gate upon checking the *Configure* button in the upper-left corner. In this scattergram, define the “compact nuclei” gate to remove irregular artifacts from the analysis by selecting the events on the upper side. If you need to select a cell line based on nuclei elongation (as in Orobeti et al. [4]), draw two gates to define the “elongated/non-elongated nuclei” on the DAPI Eccentricity/DAPI Mean intensity scattergram after choosing the “compact nuclei” gate as input. Finally, use a histogram to depict “Texa DOTS_ON_DAPI” cells signal distribution on the selected cell subpopulation in the input gate (e.g., A375, NHEM, or co-culture). An example of a gating strategy is available in Figure 8 of the article Orobeti et al. [4].

**Critical:** Use backward gating after each refining step to check if the gated events are properly identified. Left-click the gate contour, right-click on it, and select *View Backward Data For Gate*. Do this for all diagrams in the series.

17. Analyze the entire region from which the setup region is a part. For this, click on the region name in the list of samples and then right-click *Analyze* from the options drop-down menu. When the analysis is finished, the region will appear as “Processed” in the sample list.

***Note:*** *Create exclusion gates for all areas containing artifacts in the sample before initiating analysis. For this, select Add Freedrawn Exclusion from the Freedrawn Tool icon on the left-side screen.*

18. Visualize the results on the diagrams and refine cutoff values and gates, if necessary.

19. To batch process all the images, select each region to apply the same strategy and press *Analyze*. Repeat for all regions to be analyzed. Check if the events fit into the scattergrams and histograms for all samples and regions. Refine limits if necessary. Set the same axes maximum values and gate settings to all regions. For this, right-click on the diagram and select *Set max value on: x*, where x is the parameter displayed on the axis to be updated. To extend the settings to all analyzed regions, open the *Manage Diagrams* dialog window and select *Apply Diagram Set to All Samples and their Regions*. Refine gates if necessary, and batch apply the changes to all analyzed regions. Right-click on each gate and select *Propagate gate x* (where x is the gate name) and then *All Samples and their Regions* when prompted by the dialog window.

## Data analysis

Data analysis consists of two main actions:

a. Exporting the statistics from the TissueQuest software as a report.

b. Exporting raw data in Excel spreadsheet(s) to be further analyzed in GraphPad Prism to generate graphs of the results, to calculate standard deviation, and evaluate the statistical significance of the possible observed differences between experimental conditions.

We will further indicate all necessary steps to perform the analysis of the image cytometry quantification for foci analysis, similarly to data presented in Orobeti et al. [4].

1. To export statistical data computed by TissueQuest, select *Tools/Statistics Report* from the menu bar.

2. In the open *Statistics Report* window, select the input regions from the sample list and perform the following steps to export for each sample the events count (e.g., total, A375 only, and NHEM only), p-γ-H2AX mean fluorescence intensity, or percentage of p-γ-H2AX-positive cells for all cell populations of interest:

a. Choose *New Column/Global Measurements Parameters*, leave *Events Count* under the *Column Name*, and select *Events Count* under *Global Measurement*.

b. For quantifying p*-γ-*H2AX mean fluorescence intensity (MFI), choose *New Column/Predefined, pgH2AX MFI* under *Column Name*, and select predefined value unit *Mean of Mean Intensity* of *Texa* marker from the propagated gate named *compact nuclei*.

***Note:*** *To analyze only a cell line from the co-culture, choose elongated/non-elongated under Select propagated gate.*

c. For quantifying the percentage of p-γ-H2AX^+^ cells, choose *New Column/Predefined*, %pgH2AX*^+^ cells* under *Column Name*, and select predefined value unit *Percent* from the propagated gate named “pgH2AX+”.

**Figure 12.**
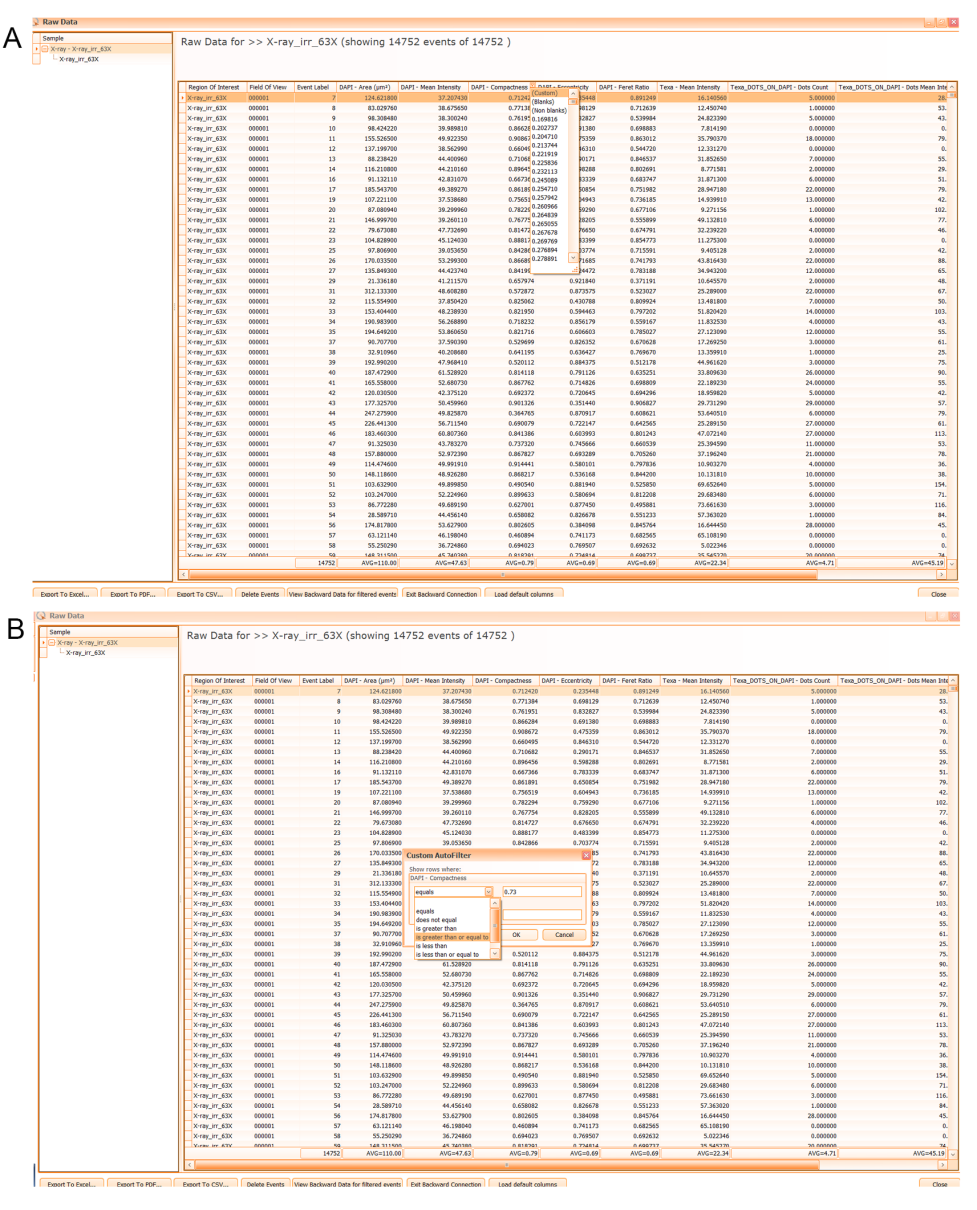
Exporting numeric values associated with the analyzed events. A. Screenshot of TissueQuest Raw Data table associated with one selected region in the sample list. B. Command to filter the events using the cutoff value. An example is given for the DAPI compactness parameter.

**Figure 13.**
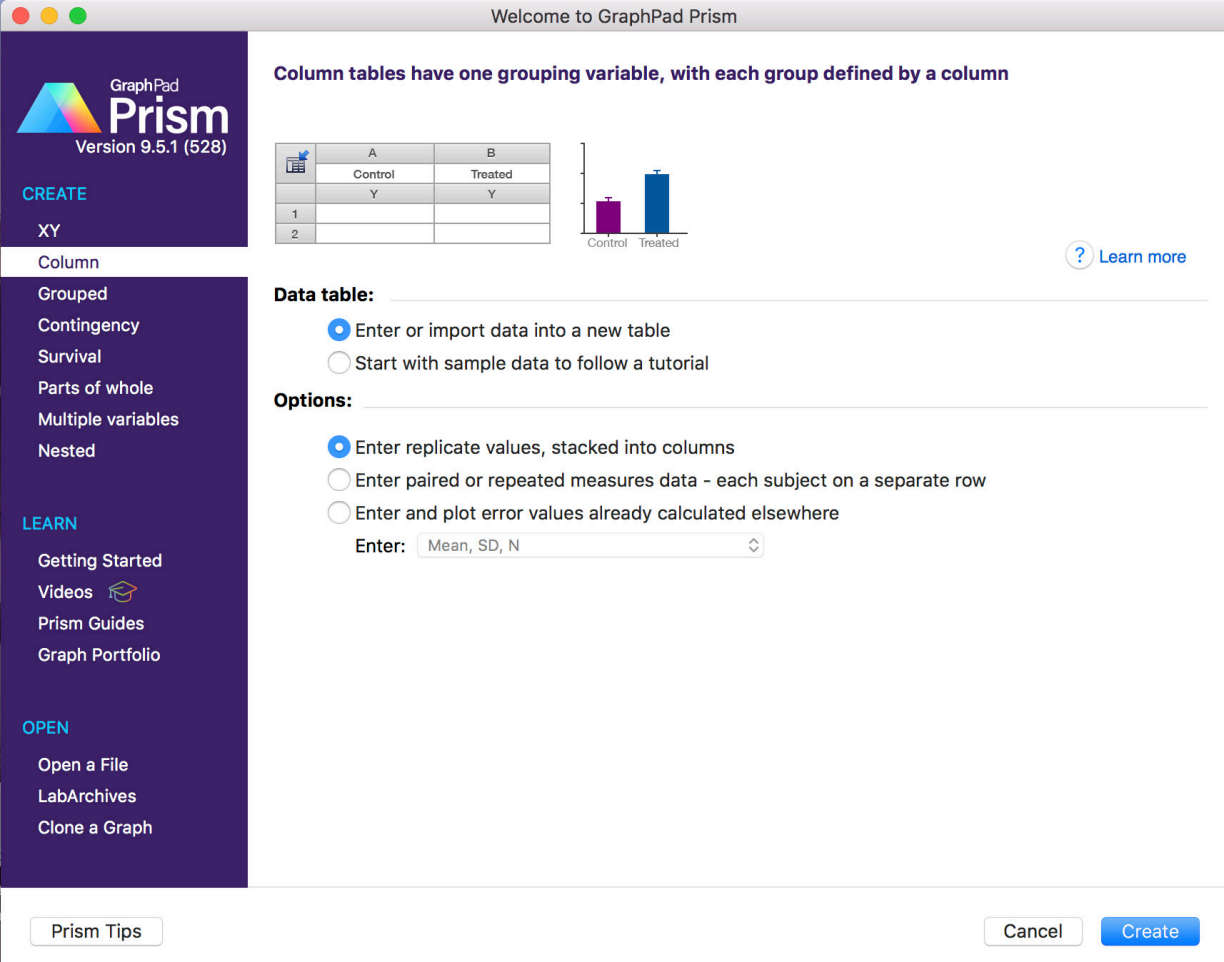
Screen capture of GraphPad dialog window. Selection of the Column graph template.

3. To export raw data for analysis into GraphPad, select each analyzed sample/region and click the *Spreadsheet* icon in the upper-right *Results* window next to *Diagram Options* and *Manage Gates* icons. In the open *Raw Data* table (see Figure 12A), filter events sequentially (using thresholds previously established in step F15) based on DAPI compactness (see Figure 12B) and DAPI eccentricity. Then, export list of the currently selected region as an Excel file (press *Export To Excel*) to extract the Texa_DOTS_ON_DAPI-Dots Count values to import into GraphPad (see next step).

4. Save the Excel file with the name of the cell line and the quantified parameter.

5. Repeat steps 3–4 to export data for all analyzed samples/regions.

6. Open GraphPad to initiate the comparative analysis of foci count for all conditions tested within the experiment. Select the *Column graph type* option (see Figure 13) that allows comparison between different (radiation) treatment conditions from the same irradiation session for “number of p-γ-H2AX foci per nucleus.”

***Note:*** *Expertise using GraphPad Prism is required to obtain the described graphs and statistical data analysis. For this, consult Prism User Guide in the Help menu tab and Prism Academy online at* *https://www.graphpad.com/prism-academy* .

**Figure 14.**
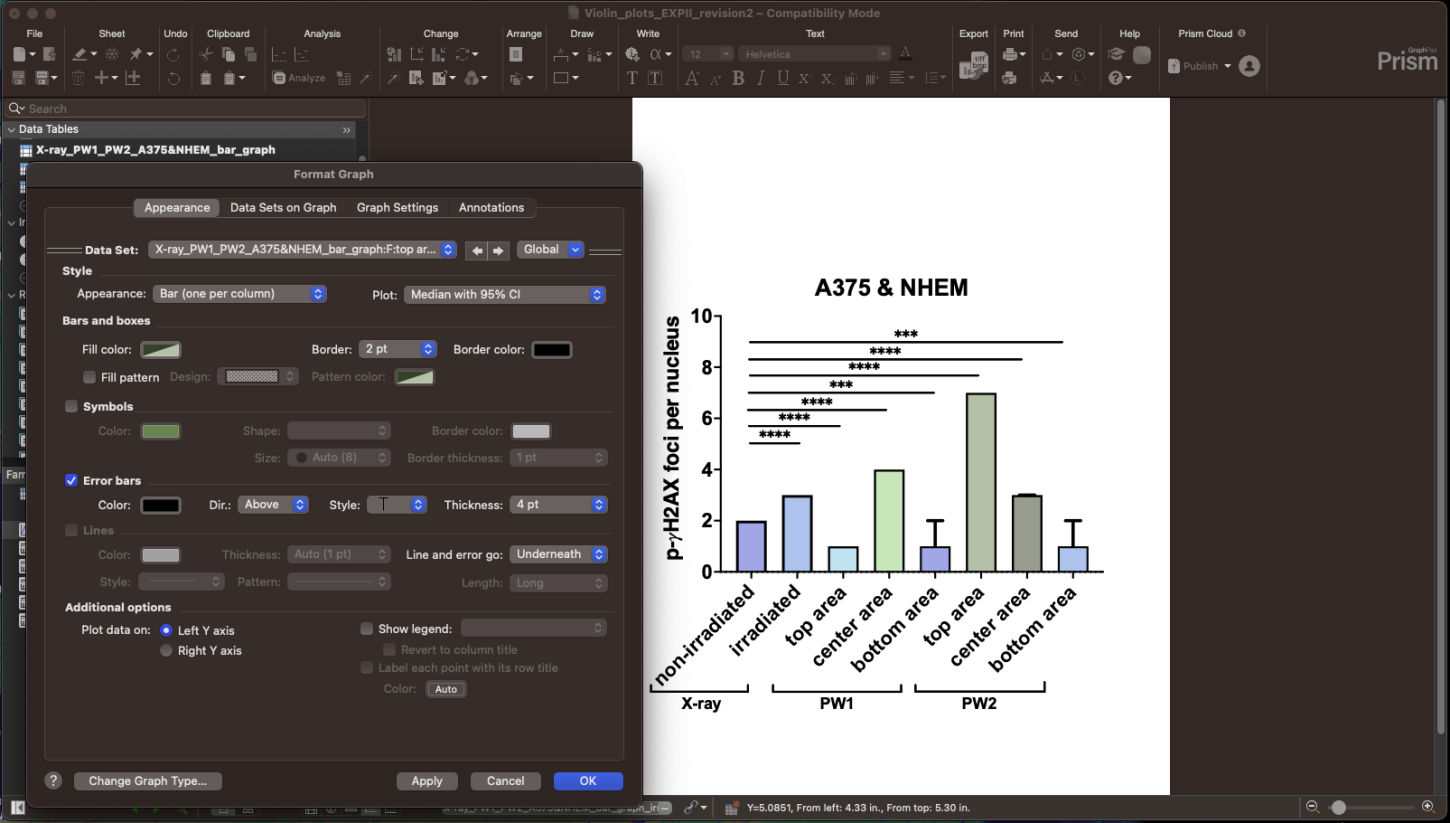
Screen capture of the *Format Graph* dialog window. Graph formatting options are visible.

7. Transfer data from Excel to GraphPad. For this, rename “Data 1” table with the name of your experimental comparison. Assign names to column titles (e.g., non-irradiated, X-ray-irradiated, etc.). Copy and paste data from each sample spreadsheet into the specific column in each table to generate graphs for co-culture (e.g., A375&NHEM) and single cultures (e.g., A375, NHEM).

8. On the autogenerated graph, double-click to refine the format (see Figure 14).

9. To evaluate potential differences in double-strand DNA breaks induction, perform statistical comparisons using a Mann–Whitney test. A p-value < 0.05 is considered to indicate statistical significance. For this, click *Analyze* in the *Analysis* tab of the menu bar. Then, choose *t tests* from the *Column Analysis* stack, select the samples for which you are doing comparative analysis, and click *OK*. Choose a two-tailed unpaired t-test without assuming Gaussian distribution followed by a Mann-Whitney test to compare ranks.

***Note:*** *If the data is parametric, a Student’s t-test may be used instead of the Mann-Whitney test. A Shapiro-Wilk test can be used in GraphPad to determine whether a data set is parametric or non-parametric.*

10. Represent the adjusted p-values on the graph for comparisons of each radiation condition with the non-irradiated control as star symbols. For this, choose *Draw* from the menu bar and select either *Manually Add Lines With Text* to select a format or *Choose Pairwise Comparisons to plot* for automatic representation of the statistical results.

11. Export the figures separately or as layout. Images should have at least 300 dpi (600 dpi for colored graphs, RGB quality) and be exported as .tiff for optimal publication quality resolution.

***Note:*** *Researchers without access to TissueQuest software can use other accessible image analysis software for foci count determination [11,12]. For this, export the “dots per nucleus” data according to the specific tool chosen and use it for statistical analysis in GraphPad (steps 6–11 presented above) or Excel.*

## Validation of protocol

This protocol has been used and validated in the following research article(s):

• Orobeti et al. [4]. First in vitro cell co‐culture experiments using laser‐induced high‐energy electron FLASH irradiation for the development of anti‐cancer therapeutic strategies. *Scientific Reports* (Figure 2, Figure 3, Figure 4, Figure 5, Figure 6, Figure 8).

## General notes and troubleshooting

**General notes**

1. For more detailed indications on the use of TissueFAXS and TissueQuest software packages, please consult the user guides in the specific software menu for updated information specific to the versions available on your system or the respective printed versions received at installation.

**Troubleshooting**

Problem 1: Samples present artifacts.

Possible cause: Coverslips or slides were not cleaned; buffers contained aggregates; use enough mounting to prevent sample drying and air bubbles formation.

Solution: A) (wet lab procedures) Pass the coverslips through MilliQ ultrapure water using fine forceps before mounting on the slide to eliminate dust particles and aggregates and to prevent PBS crystals from forming. Clean the slide with 70% ethanol using Kimwipes. Filter buffer solutions used for immunofluorescence staining. B) (software procedure) Draw exclusion areas around the artifacts to remove them from the analysis. Any area with a different background level within the sample induces threshold errors in the analysis, thereby affecting the quality of the quantitative analysis results. Therefore, it should be removed.

Problem 2: Nuclei segmentation results are not precise.

Possible cause: Signal-to-noise ratio is deficient; DAPI analysis parameters are not optimal.

Solution: A) (wet lab procedures) Prevent background by carefully and thoroughly washing the samples after each staining step and by not using excessive mounting solution. B) (software procedure) Also, carefully optimize the exposure time before sample scanning to obtain a bright signal without increasing background levels. To optimize nuclei masks during segmentation, fine-tune background threshold parameters until the masks overlap with the desired signal. To observe background gray levels, hover the mouse over the space between nuclei, and the value will be shown in the upper-left corner of the image. The lower signal threshold level should be above that of the background. Finally, if two nuclei are seen as one, a line can be used to manually separate them to optimize correct segmentation; alternatively, exclude doublets and aggregates during analysis when applying the first gate using DAPI Area measurement.
